# Patient Safety Culture in European Hospitals: A Comparative Mixed Methods Study

**DOI:** 10.3390/ijerph19020939

**Published:** 2022-01-14

**Authors:** Nina Granel-Giménez, Patrick Albert Palmieri, Carolina E. Watson-Badia, Rebeca Gómez-Ibáñez, Juan Manuel Leyva-Moral, María Dolors Bernabeu-Tamayo

**Affiliations:** 1 Grup de Recerca Infermera en Vulnerabilitat i Salut (GRIVIS), Department d’Infermeria, Universitat Autònoma de Barcelona, Avda. Can Domènech, Edifici M. Despatx M3/213, Bellaterra, 08193 Barcelona, Spain; nina.granel@uab.cat (N.G.-G.); rebeca.gomez@uab.cat (R.G.-I.); juanmanuel.leyva@uab.cat (J.M.L.-M.); mariadolors.bernabeu@uab.cat (M.D.B.-T.); 2 Department d’Infermeria, Facultat de Medicina, Universitat Autònoma de Barcelona, Avda. Can Domènech, Edifici M. Despatx M3/213, Bellaterra, 08193 Barcelona, Spain; carolina.watson@uab.cat; 3 Evidence-Based Health Care South America: A JBI Affiliated Group, Calle Cartavio 406, Lima 15023, Peru; 4 South American Center for Qualitative Research, Universidad Norbert Wiener, Avenida Arequipa 440, Lima 15046, Peru; 5 Doctor of Health Sciences Program, College of Graduate Health Studies, A. T. Still University, 800 W. Jefferson Street, Kirksville, MO 63501, USA; 6 Center for Global Nursing, Texas Woman’s University, 6700 Fannin Street, Houston, TX 77030, USA; 7 Knowledge Management Department, Hospital Universitari Vall d’Hebron, 08035 Barcelona, Spain

**Keywords:** patient safety, organizational culture, safety management, safety culture, hospital survey on patient safety culture, adverse events, nursing, public health, hospitals

## Abstract

Background: Poorly organized health systems with inadequate leadership limit the development of the robust safety cultures capable of preventing consequential adverse events. Although safety culture has been studied in hospitals worldwide, the relationship between clinician perceptions about patient safety and their actual clinical practices has received little attention. Despite the need for mixed methods studies to achieve a deeper understanding of safety culture, there are few studies providing comparisons of hospitals in different countries. Purpose: This study compared the safety culture of hospitals from the perspective of nurses in four European countries, including Croatia, Hungary, Spain, and Sweden. Design: A comparative mixed methods study with a convergent parallel design. Methods: Data collection included a survey, participant interviews, and workplace observations. The sample was nurses working in the internal medicine, surgical, and emergency departments of two public hospitals from each country. Survey data (*n* = 538) was collected with the Hospital Survey on Patient Safety Culture (HSOPSC) and qualitative date was collected through 24 in-depth interviews and 147 h of non-participant observation. Survey data was analyzed descriptively and inferentially, and content analysis was used to analyze the qualitative data. Results: The overall perception of safety culture for most dimensions was ‘adequate’ in Sweden and ‘adequate’ to ‘poor’ in the other countries with inconsistencies identified between survey and qualitative data. Although teamwork within units was the most positive dimension across countries, the qualitative data did not consistently demonstrate support, respect, and teamwork as normative attributes in Croatia and Hungary. Staffing and workload were identified as major areas for improvement across countries, although the nurse-to-patient ratios were the highest in Sweden, followed by Spain, Hungary, and Croatia. Conclusions: Despite all countries being part of the European Union, most safety culture dimensions require improvement, with few measured as good, and most deemed to be adequate to poor. Dimension level perceptions were at times incongruent across countries, as observed patient safety practices or interview perspectives were inconsistent with a positive safety culture. Differences between countries may be related to national culture or variability in health system structures permitted by the prevailing European Union health policy.

## 1. Introduction

The European Union, or EU, has 28-member countries with similarities in the public health systems due to common regulations but different approaches for financing and delivering health services to their populations [1]. Health systems in the EU are products of the political, cultural, and socioeconomic traditions of each country [2]. There are 11 countries with national health systems providing services to citizens through direct income tax payments, which is the Beveridge model [3], while the other 17 countries support social health insurance systems under the Bismarck model [4], where people pay fees to fund their health services. As a result, the implementation of strategies to improve health system outcomes and decrease failures such as adverse events vary widely across the EU [5]. Adverse events result in 8% to 12% of EU hospitalizations [6] with a significant global burden [7]. Reducing the impact of adverse events in European hospitals requires organizational strategies with a multidisciplinary approach in which nurses have an important role [8]. Despite an early record of robust legislative achievements in patient safety [9], the European Union has produced no new regulations for nearly a decade [10].

### 1.1. Background

Twenty years ago, the seminal report To Err is Human [11] exposed a hidden patient safety crisis by identifying at least 99,000 patient deaths each year in the United States due to preventable medical errors. Shortly thereafter, researchers began linking hospital clinical quality and patient safety outcomes to organizational properties [12] such as communication, leadership, mutual respect, and teamwork [13]. For this reason, international organizations such as the World Health Organization (WHO), national agencies such as the United States Agency for Healthcare Research and Quality (AHRQ), and accreditation organizations such as The Joint Commission recommended that hospitals immediately begin the work to improve hospital organizational cultures.

Despite a large body of work to reduce medical errors, as many as 16% of hospitalized patients still experience a medical error [14], with adverse events representing the third leading cause of death in the United States; estimates range between 250,000 [15] and 440,000 [16]. The patient safety problem is consequential for patients and expensive for health systems [7]. Although unsafe practices are a major error source [17], errors remain hidden without robust error reporting. For this reason, organizations need to be preoccupied with failure and committed to being resilient [18] as they respond to errors with strategies focused on improving processes rather than punishing people [19].

### 1.2. Patient Safety Culture

According to the WHO [20], patient safety is the ‘prevention of errors and adverse effects to patients associated with health care.’ Patient safety is derived from an organizational culture [21] and the collective behaviors of individuals. As such, patient safety culture, or safety culture, can be broadly described as the summative impact of activities such as individual and group values, attitudes, perceptions, competencies, and patterns of behavior in pursuit of this goal [22]. The leaders ultimately determine the organizational commitment to patient safety and proficiency for safety management as they orchestrate the opportunity to learn from mistakes [23,24]. For this reason, leaders seek to strengthen system resilience [25] to medical errors with targeted quality improvement strategies [26] to improve the hospital culture by supporting the patient safety practices of everyone.

The safety culture of a hospital can be assessed with instruments designed to measure safety properties such as management and supervision, safety systems, risk perceptions, teamwork, communication, feedback, reporting systems, workload, personal and psychological resources, and other organizational factors [27]. Applied in more than 20 different languages across the world [28], the AHRQ Hospital Survey on Patient Safety Culture [22], is the most commonly used instrument to measure safety culture in the health sector. The survey assesses the effectiveness of the work environment by measuring the organizational process and clinical practices associated with preventing the types of errors linked to consequential adverse events [13,29]. When administered yearly, the survey offers leaders a proxy measure for the effectiveness of their improvement efforts focused on achieving patient safety [30].

### 1.3. Purpose of the Research

Robust study designs incorporating surveys, interviews, and observations are essential for researchers to understand safety culture in greater depth through contextual evaluation and comparative analysis [31,32]. Yet, there is only limited evidence from Iran [33] and Ethiopia [34] resulting from mixed methods studies focused on patient safety culture [35], with no multinational comparative studies reported in the literature. As such, the purpose of this study was to measure the safety culture perceived by nurses, the largest segment of health professionals, working in public hospitals in four European Union countries. The principle objectives were to: (1) measure the safety culture perceived by nurses working for public hospitals in four countries; (2) observe the safety practices of hospital nurses working for public hospitals in four countries; (3) identify strengths and weaknesses of the safety culture perceived by nurses working for public hospitals in four countries; and (4) compare the safety culture perceived by nurses working for public hospitals in four countries with observed practices and lived experiences. 

## 2. Materials and Methods

### 2.1. Study Design

This was a mixed methods study with a convergent parallel design [36] following the process outlined by Creswell and colleagues [37]. The integrated design allowed the research team to maintain the study fidelity with two sequences including quantitative measurement and a qualitative assessment [38]. The findings from the two segments were unified and triangulated to provide an overall interpretation of the data [39].

### 2.2. Setting and Sample

The study was conducted in public hospitals in four European Union countries including Croatia, Hungry, Spain, and Sweden. The sample included professional nurses working in internal medicine, general surgery, and the emergency units of eight public hospitals, two units from each country. The selected units had the highest percentage of nurses commonly employed across the hospitals in the selected countries. All nurses working in the hospital for a minimum of one year were eligible to participate in the quantitative data collection; there were no exclusion criteria. Interview participants were purposively selected using a critical case sampling approach to identify nurses most likely to provide the most useful information. The inclusion criterion was three years of work experience in the unit; efficient and expert nurses according to Benner’s [40,41] novice to expert model. The goal was to gain a deeper understanding about the nursing care, clinical practices, and the working conditions related to the twelve safety culture dimensions.

### 2.3. Data Collection

Once the hospital research and ethics committees approved the study, the data collection process advanced through the quantitative and qualitative sequences. The quantitative sequence of data collection included administration of the Hospital Survey on Patient Safety Culture (*HSOPSC*), while the qualitative sequence included the ethnographic processes of work observation and participant interviews.

### 2.4. Quantitative Sequence

The survey was administered in a sequential process by country; Hungary (February 2016), Croatia (Abril 2016), and Spain (April 2017). For the two hospitals in Sweden, secondary data reported from the national HSOPSC survey was used for analysis. This resulted in not asking the staff to repeat the same survey twice within the same year. For the original data collection, the primary investigator presented the project to the nursing unit supervisors, and they distributed the survey to the nursing staff. The completed surveys were anonymously deposited into a secured box on each unit. To increase the response rate, weekly reminders were made by the supervisors and the principal investigator to the nurses working in each shift during the data collection period. 

The HOSPSC has demonstrated excellent psychometric properties [42,43], with validated versions used throughout the world [44], including the countries in this study [45,46,47,48,49]. The HSOPSC contains 42-items organized by 12 dimensions with two types of 5-point Likert scales [50]. Nine dimensions have items with perceptions measured on a 5-point Likert scale to indicate level of agreement (“strongly disagree” to “strongly agree”), and three dimensions have items with five perceptions for frequency (“never” to “always”). The survey also measures demographic information, patient safety grade, and number of incidents reported in the previous year. 

The main outcome for this study was the level of positive perception for each dimension, which was calculated by the number of positive perceptions for each item in a dimension divided by the total number of perceptions for all items. The negatively worded items were recognized for reverse coding prior to calculation. An organization, department, or unit demonstrates an ‘excellent’ safety culture (survey, dimension, or item) with a positive perception ≥90%, a ‘good’ safety culture with a positive perception between 70% and 89%; an ‘adequate’ safety culture with a positive perception between 50% and 69%; and a ‘poor’ safety culture with a positive perception lower than ≥49%. For the analysis cut point in this study, a positive perception with an average score ≥ 75%, was defined as an area of strength while an average score ≤ 50% was defined as an area of weakness.

### 2.5. Qualitative Sequence

Simultaneous with the quantitative sequence, original qualitative data was collected in Croatia, Hungary, Spain, and Sweden through 24 (six in each country) in-depth semi-structured interviews. In addition, the primary investigator observed the daily work of nurses on each unit for about 150 h (approximately 37 h in each country). For the observations, the principal researcher was introduced to the nurses at the start of each shift. To minimize the Hawthorne effect [51], the researcher wore an institution uniform to blend into the unit activities and willingly answered questions from the nurses. The individual performance, work routines, and actions were observed, and field notes were recorded. Dynamics related to teamwork within the unit were also observed. 

**Figure 1:**
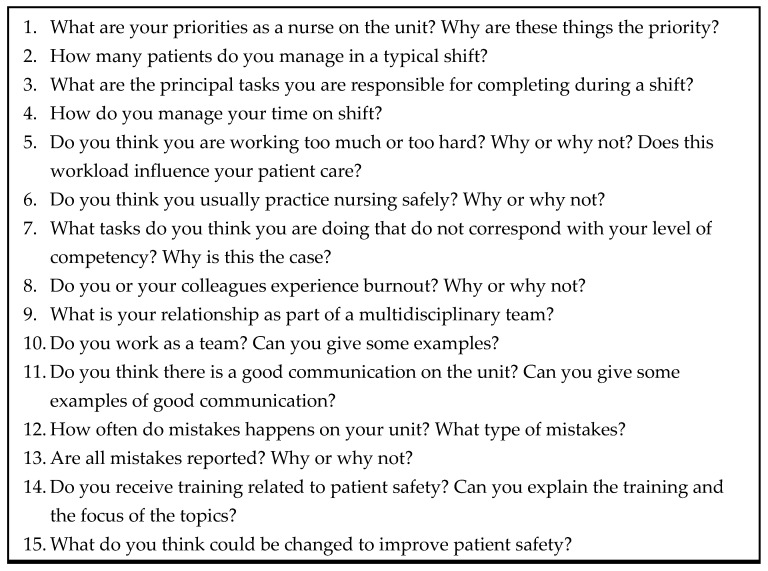
Semi-structured interview guide.

During the non-participant observations, the primary investigator purposefully invited nurses to voluntarily participant in interviews outside the work area; according to their preference, in a meeting room provided by the hospital. Interviews were conducted in English or Spanish, with each interview lasting between 30 to 50 min. In the case of Croatia and Hungary, a nursing student with an advanced English certificate participated as a translator, when necessary, to accommodate the English level of the nurses. The interviews were digitally recorded until data saturation was reached following a semi-structured interview guide (Figure 1). The Spanish audio-recorded interviews were transcribed verbatim and translated into English by the principal researcher.

### 2.6. Data Analysis

Quantitative data were analyzed using SPSS 23.0 (IBM Corp.: Armonk, NY, USA) for descriptive and inferential statistics. The significance level for the bilateral contrasts was *p* ≤ 0.05. The categorical variables were summarized with absolute and relative frequencies, and the continuous variable with means and standard deviations. Pearson’s chi-squared test was used to test proportions, and variance analysis with Tukey’s post-hoc correction or Dunnett’s T3 was used to test means, depending on variance homogeneity. 

Qualitative data, including participant responses to interview questions and field notes for observations, were analyzed using Atlas.ti software, version 7.5.12. (ATLAS.ti Scientific Software Development GmbH: Berlin, Germany). Content analysis was used to code and categorize the data guided by the twelve dimensions of patient safety culture [52]. Data were coded by country (CR—Croatia, HU—Hungary, SP—Spain, and SW—Sweden), collection technique (OBS—non-participant observation, and INT—in-depth interview), individual participant numbers, and nursing units (MED—medicine, SURG—surgery, and EMERG—emergency).

A spider-web diagram was developed as a visual presentation of the twelve dimensions by country to guide the triangulation of data: survey, observation, and interview. The diagram provides the opportunity for rapid dimension assessments, including comparison by country. Importantly, the diagram provides an easy method for identify strengths and weaknesses to integrate qualitative data into the comparative analysis. The spider-web diagram is an essential tool for organizing large quantities of diverse data derived from a mixed methods study for a comparative analysis.

### 2.7. Rigor and Trustworthiness

Strategies to ensure methodological rigor of the quantitative methods [53,54] included a qualified research team, strategic planning, adherence to a defined protocol, use of validated instruments, adequate sample size, and reliable data analysis. Trustworthiness [55,56] of the qualitative data was demonstrated with the use of a semi-structured interview guide, documented audit trail, research team reflexivity, open communication, and the achievement of data saturation.

## 3. Results

A total of 650 questionnaires were distributed to nurses in the participating countries, and 538 were completed (Croatia *n* = 60, Hungary *n* = 151, Spain *n* = 109, Sweden *n* = 218). The overall response rate was 89% (each country > 72%). Overall, 401 nurses (74.5%) had more than five years work experience. Sweden 179 (82.1%) and Spain 91 (83.5%) had the most experienced nurses, while Hungary 87 (58%) and Croatia 44 (74.5%) had significantly less experienced nurses (*p* < 0.001). The number of staff working more than 40 h per week was 53 (88.3%) in Croatia, 81 (53.6%) in Hungary, 27 (24.8%) in Spain, 81 (37.2%) in Sweden; significantly different between countries (*p* < 0.001). 

**Figure 2:**
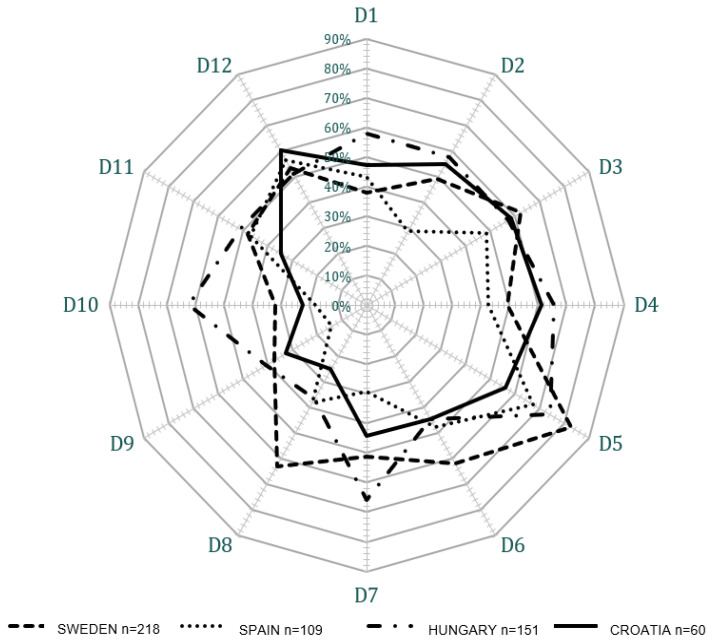
Positive safety culture perception for each HSOPSC dimensions by country.

Due to the complexity of the data for the twelve safety culture dimensions measured across four countries, including eight hospitals, the data country level survey data were organized into a concise representation for comparison, interpretation, and triangulation with the qualitative data. The spider-web diagram (Figure 2) provides the simplified visual presentation of the survey data for each of the twelve dimensions by country.

In the context of the detailed results, Table 1 provides a comparison of safety culture dimensions across countries and between hospitals. The hospital level survey data for Croatia (hospital 1 = 28, hospital 2 = 32), Hungary (hospital 1 = 87, hospital 2 = 64), Spain (hospital 1 = 63, hospital 2 = 46), and Sweden (hospital 1 = 130, hospital 2 = 88) are reported by average positive perception for each safety culture dimension. There are multiple statistically significant differences noted between countries with fewer noted within country. The complete unit level comparison of positive perspectives for each safety culture dimension is presented in Table 2. Except for three instances, the dimensions between hospital units within country were not statistically different. Interview data contributed evidence across all dimensions for each country, but only the dimensions considered to be strengths or those considered to be weaknesses were identified for further analysis. 

### 3.1. Dimensions of Strength

The highest positive perceptions across all the countries were identified for dimension 5 (teamwork within units). This was the only dimension with a positive perception greater than 75%, reflecting a strength for multiple countries. In Sweden, teamwork within units was considered a strength for all hospitals and within each unit (82.7%). The observations evidenced the staff practices and routines resulted in teamwork; this was also corroborated by the interviews.

*The nurse and the assistant nurse work as a team, all day, and we check out with each other what we have to do, and what we do next, and so on. *

*(SW, INT-16, MED)*

*I mean we communicate a lot with the doctors, but many other things. And also, if we have a patient, that has breathing problems, I know that the doctor wants an arterial blood gas, so then I just take it, at once, because I know he wants it, so. We are quite independent, yeah, we talk a lot as well with each other, yeah. *

*(SW, INT-13, EMERG)*

*We sit together and everything, so we always sit together with the doctors, so that’s good for the communication, very good. *

*(SW, INT-14, EMERG)*

In Spain, staff generally supported each other, and the teamwork between nurses and nursing assistants was evidenced in all the data sources. Dimension 5 had high positive perceptions for all hospitals and units (67.4%, 34.3) but was only considered a strength for the surgical units (83.1%, 25.2, *p* < 0.001). From the qualitative data, there were some challenges among nurses with physicians but good relationships with the nursing assistants.

*The communication with healthcare assistants is good; we delegate work to them and…there is no problem. *

*(SP, INT-6, SURG)*

*One nurse on shift asks other nurses and healthcare assistants if they have finished the patients’ hygiene rounds and if they needed help. *

*(SP, OBS-3, OBS-4, MED, and SP, OBS-2, OBS-6, SURG)*

*The medical office is next to the nursing office, and that helps a lot. It contributes to good communication with the doctors, but it also depends with whom you communicate better, you know, who to address to. *

*(SP, INT-6, SURG)*

In Hungary and Croatia, dimension 5 (teamwork within units), had also high percentages, (74.0%, 28.4) and (55.9%, 35.8) respectively, but was only a strength for the Hungarian medical units (77.6%, 25.6). In both countries, elements of teamwork among nurses were detected through the interviews, however, the observations resulted in some discrepancies with the physicians. These relationships were cordial but seemingly hierarchical with limited interactions.

*We (the nurses) have no other possibilities due to the amount of work, we must work together as a team. *

*(HU, INT-5, SURG)*

*The friends always work together; because they always understand each other, and the head nurse makes the schedule and put friends together it is better for the patients and for everybody. *

*(HU, INT-4, MED)*

In the Croatian emergency units, observations evidenced the nurses worked as a team; the positive response was higher than other units. However, one participant described a clearly subservient relationship of nurses with physicians.

*Our nurses depend to doctors, we are like servants of doctors, and we do not have the idea that we can be a profession, we have to professionalize nurses. *

*(CR, INT-2 EMERG)*

### 3.2. Dimensions of Weakness

In Sweden, dimensions 1 (frequency of event reporting), 9 (staffing) and 10 (hospital management support for patient safety) were clearly perceived by the nurses to need improvement. Three other dimensions had percentages approaching 50%, including dimension 2 (overall perceptions of safety), 4 (organizational learning—continuous improvement), and 11 (teamwork across hospital units), see Table 1 and Figure 2.

The interview data indicated nurses perceived a lack of support from hospital management resulting in poor work conditions including the lack of staff and excessive work pressure. In terms of observed staffing, the ratio in the surgical and internal medicine units were approximately one nurse for every five patients with the units having nursing assistance on each shift. However, the nurses indicated they are working too many extra shifts.

*We are short of staff. There’s a lot of people that is taking extra shifts and working in double shifts. *

*(SW, INT-15, MED)*

During the observations, pressure on shift was not generally visible. In this regard, the nurses had time for meals and breaks. One participant considered burnout syndrome to be common; however, this was not generally reported by all the participants.

*I don’t know if I should say this, but it’s usually the same people that complain. But mostly the staff that take this extra shifts so… yeah, some people burnout. *

*(SW, INT-15, MED)*

Swedish nurses acknowledged things were sometimes forgotten and that misunderstandings could result. According to the nurses, the response to a mistake was generally not punitive. Yet, one nurse indicated mistakes were often not reported due to time constraints.

*Yes, it’s very very tough sometimes, very very tough, because, you know, so many patients and we don’t have time and we just run around and doing things and… So it’s easy to miss something. *

*(SW, INT-13, EMERG)*

*Of course, mistakes happen, but not very often perhaps it’s more like… well, if we, in the rounds with the doctors, something is decided with the patient, and… sometimes perhaps we have no time because we have so much other things to do and it’s hard to prioritize sometimes, and it can be perhaps a lack of understanding, or misunderstanding. *

*(SW, INT-15, MED)*

*There comes the time, I think many small mistakes don’t get reported. Because the lack of time. You choose to take care of the patient instead of filling in this report. *

*(SW, INT-16, MED)*

In Spain, all the dimensions with the exceptions of 5 (teamwork within hospital units) and 12 (handoffs and transitions) were perceived by the nurses to require improvement (see Table 1). These results were triangulated by interview and observational data. In terms of staffing, the ratios in the surgical and internal medicine units were observed to be approximately one nurse for every ten patients. Units also had nursing assistants on each shift. Nurses unanimously reported there were not enough staff for patient safety as they often worked in ‘crisis mode’ trying to do too much, too quickly. Excessive concerns about safety were reported as well as observed related to the lack of time to complete important tasks, and wellness due to irritability, tension, and fatigue. The qualitative data indicated nurses generally believed patient safety could be improved but the improvements were limited by the lack of resources. Overall, the work pressures experienced by the nurses placed them at risk for making errors.

*Always, there is always pressure, if not the doctors, the relatives … the workload … it is a lot. If you want to do things right, it is a lot. *

*(SP, INT-3, MED)*

*There is work pressure in some shifts with less staff. The workload is the same, and supervisors can see that you can’t manage it, and you ask for extra staff and they don’t arrive, perhaps because nobody’s available or because it’s not possible at that moment. *

*(SP, INT-2, SURG)*

*[You go home thinking] thank goodness, I’ve finished, because I would have caused a disaster. Often you leave with that feeling, or the feeling that you have forgotten things because you don’t have enough time! *

*(SP, INT-6, SURG)*

Most nurses in Spain associated ‘incidents’ only with medication errors and verbalized that, sometimes, they discussed these with the physicians. However, neither physicians nor nurses reported these incidents through the formal reporting process.

*I think that the majority get notified, but all? I don’t think so. If you realize, you tell the doctor; but, it doesn’t go any further. *

*(SP, INT-4, MED)*

*Once, a nurse made a mistake; she administrated three 1 mL morphine ampoules when she had to administer 0.3 mL. She told the doctor, but they did not report it. *

*(SP, INT-6, SURG)*

There was a diversity of perspectives and some discomfort among the nurses when speaking about detecting errors. The nurses believe the lack of reporting was related to perceived culpability when incidents occur and the fear of punishment.

*Well, I guess it’ll be for fear of reprisals or for … the person above you … you know, he can blame you in some way or, if it’s not that, what other reason can there be? If nothing happens, no one finds out. *

*(SP, INT-3, MED)*

The emergency units had lower positive perspectives for most dimensions (10 of 12), with significant differences for dimensions 4 (organizational learning and continuous improvement) (*p* = 0.007) and 5 (teamwork within hospital units) (*p* < 0.001); see Table 2. This unit has many staff, with observations about limited communication and interactions not aligned with a good safety culture. 

In Hungary, the dimension 6 (communication openness), 8 (non-punitive response to error), and 9 (staffing) were perceived by nurses to need improvement (see Table 1). In terms of staffing, the observed ratio in the surgical and internal medicine units were approximately one nurse (diploma or graduate) for every ten to twelve patients. The nurses were generally observed to have time for short breaks. All the nurses referred to the lack of staff as the major contributor to high levels of work pressure. Some nurses believed the pressure directly contributed to less time to care for their patients.

*Also, the time to care for one patient is also not enough, they have not enough time to treat, to care the patients. *

*(HU, INT-6, SURG)*

*For example, the demands are very high for the staff. Because of it they are tired, weak, and sometimes impatient with the patient. *

*(HU, INT-4, MED)*

However, some staff indicated work pressure on the united considered did not impact their patients.

*In the unit there are lot of pressure. The nurses has a staff room for breaks, so the pressure does not put at risk the patient. *

*(HU, INT-3, MED)*

*The unit is always full, every bed is occupied. It means physical pressure and psychological pressure for the workers, but no, it is impossible. The patient does not feel the staff pressure. No. *

*(HU, INT-5, SURG)*

Generally Hungarian nurses indicated during the interviews there are rarely mistakes, and in the rare case there was a mistake, this was communicated to the physician and the supervisor. They believe the mistakes are addressed rather than hidden.

*I do not mean that everything is perfect in the unit. Sometimes happen small mistakes or small problems. We have selective waste bin collection and sometimes somebody take the waste not to the right place. Sometimes staff do not perform the right alcoholic hand disinfection. *

*(HU, INT-5, SURG)*

*It is the same than in other departments, if there is a mistake, the nurse tells the head nurse, and they write everything on the computer if there is an unexpected event. *

*(HU, INT-3, MED)*

*If a staff member recognizes a mistake, they have to tell immediately the head nurse and the doctor. If the dose is not correct [referring to medication administration] they report it. It is impossible to say I will hide it and do not speak about it. *

*(HU, INT-6, SURG)*

*If we make a mistake, we report it immediately and if there is a fall of the patient as well. *

*[HU, INT-4, MED)*

The hospital responses to mistakes were clearly punitive, having direct consequences to the staff.

*If staff make a professional mistake, it has consequences, but I do not know any professional mistake. If the patient problem comes from the staff’s mistake and it is proved that the problem was the staff’s mistake, it will mean legal consequences. For example, they give a warning to the nurse if she makes a mistake. And she has to participate in training. If something happens in the unit, they have to speak about it to avoid it happening again. *

*(HU, INT-6, SURG)*

In Croatia, there were seven dimensions perceived by the nurses to require improvement (see Table 1). These dimensions included, 1 (frequency of event reporting), 6 (communication openness), 7 (feedback and communication about error), 8 (non-punitive response to error), 9 (staffing), 10 (hospital management support for patient safety), and 11 (teamwork across hospital units). In terms of staffing, the observed ratio in the surgical and internal medicine units were approximately one nurse (diploma or graduate) for every 10–15 patients. Most participants mentioned that they frequently worked short staffed, resulting in fatigue and burnout. The lack of staff was also believed to negatively impact the quality of patient care.

*I often work as a triage nurse, we do 12 h shift, when we do it 3 or 5 times a week it is too heavy, I feel pressure, because you must communicate with patient, with family, other staff, answer the telephone. And I think that it is difficult, and some nurses get burnout. *

*(CR, INT-2, EMERG)*

*We do not have enough nurses, it is the big problem, the hospital does not have money to employ more nurses. I was working with 30 patients in the surgical unit, we cannot provide good care because we do not have enough time. *

*(CR, INT-4, EMERG)*

Most nurses commented about the authoritative approach of supervisors who manage their units. They believed the approach results in disrespectful interactions and unilateral communications to the staff. As a result, the staff are unwilling to engage in open communication about problems.

*The communication sometimes is not good. Sometimes can be good, but sometimes…I do not have words, it is difficult. Ward sister [supervisor] will tell everything to a young sister and sometimes not with such great words, sometimes rude, without good manners. *

*(CR, INT-6, SURG)*

*Yes, sometimes we forget to tell things to the doctor. Sometimes we have patient with the same name, there happened in the past to take the wrong patient to Xray. We have problems of communication when it is very busy in the ward, when we have lots of patients, and we are so nervous, can be a problem, sometimes. *

*(CR, INT-2, EMERG)*

Few nurses in Croatia verbalized a different perspective regarding patient safety during the interviews, indicating there was very good communication, sufficient staffing, and no processes or procedures that put patients at risk. They also indicated there are rarely mistakes on their units.

*No, there is enough staff, they do not need to hurry, so they always double check and almost never mistakes happened. System and protocols work well. They have a lot of patients, but they manage to work with them. Mistakes here aren’t, they cannot happen, mistakes are very rare. The main doctor and nurse manager know everything! *

*(CR, INT-1, EMERG)*

*We have meetings for the staff every month, we can speak freely what we like what we do not like. *

*(CR, INT-2, EMERG)*

*Is very rare [mistakes]. Because they [nurses] write what has to be done tomorrow in the morning. Every nurse knows everything about the patient. *

*(CR, INT-3, MED)*

However, the response to the rarely occurring errors was perceived to be very punitive. Two nurses from the surgical and emergency units stated they are afraid to speak freely; the behavior is related to the cultural context that causes anxiety and stress.

*I, for example, if I do not give a pill, everybody will know. If you make a mistake, you are not good for the rest of your life…Our population do not say the truth, it is our mentality, our culture, better to say a lie, to have better and nice relationship with others, better to lie that say the truth and cover yourself. You do not have to know everything but I will tell them everything about you but you do not have to know that, it is our thinking. I hate it, really hate it. We have to be crazy to do this job, if you are a good person here, you will suffer. *

*(CR, INT-6, SURG)*

*I have not been here for a long time, I can see [problems] and must be quiet. I cannot identify mistakes. Sometimes we do not tell her [supervisor] everything, and that is our problem, we probably have to say everything, what it is going on in this unit, but sometimes we do not. *

*(CR, INT-2, EMERG)*

## 4. Discussion

This study provides an evaluation of the safety culture perceived by nurses working in public hospitals in four European countries. The data was presented in comparative terms with dimensions defined by strengths or weaknesses, but each dimension can also be assessed as excellent, good, adequate, and poor perceptions. With the opportunity for data triangulation resulting from the mixed methods design, the additional qualitative context provided a deeper understanding about the perceptions through comparative analysis. For this reason, the quantitative findings can be discussed in the broader context of the nurse interviews and work observations for comparison to their perceptions.

The positive perception of safety culture for each HSOPSC dimension by country was perceived to be ‘adequate’ by the majority of Croatian, Hungarian, and Swedish nurses in this study and almost ‘adequate’ for Spanish nurses. Previous studies from Croatia [45], with nurses and physicians (*n* = 561), and Spain [57], with health care professionals (*n* = 6257), reported similar results. However, the work observations of nurses, and the experiences shared by the nurses in each country, indicate that the definition for ‘adequate’ may not be consistent across countries. Despite the health expenditures being higher in Spain, with more staffing and technology, than in Hungry and Croatia, the overall perception of ‘good’ and ‘adequate’ were more often observed in these Eastern European countries. 

Nurse staffing and workload pressures were identified as areas requiring improvement across the counties. Previous research indicates that staffing is associated with the workplace conditions resulting in patient safety [58]. An earlier study by Halbesleben and colleagues [59] also found an inverse relationship between burnout and patient safety indicators including a reduced safety perception by nurses experiencing burnout. This finding is consistent with the results across the four countries in this study.

Nurse-to-patient ratios influenced nurse perceptions about safety culture at the unit level and across the organization. The relationship between workload associated with nurse-to-patient ratios and patient safety outcomes has been well-established [60]. There are some contradictions in the literature; however, in relationship to the longevity of work experience in the same institution. In this regard, some studies indicate that more tenure provides more critical comments and negative responses while other studies are contrary [61]. Similarly, the finding between countries in this study were contradictory, with a clear discrepancy in the quantitative measurement of dimension 9 (staffing) as the highly staffed units in Sweden were perceived by Swedish nurses to be nearly the same as the much lower staffed units in Hungary by Hungarian nurses.

Nurses in Spain perceived working in ‘crisis mode’ as they attempted to do too many things in the shortest possible time due to inadequate staffing (dimension 9, staffing). The visibly accelerated work pace resulted in multiple physical consequences such as irritability, muscle tension, and fatigue observed in this study. Importantly, these are classic signs of unhealthy stress in the workplace [62,63]. The Spanish nurses perceived staffing to be poorest of the twelve dimensions as well as the lowest staffing dimension across all the countries. Although the Croatian and Hungarian nurses were more poorly staffed in comparison to Spain and very poorly staffed in the context of Sweden, their positive perceptions about staffing were surprisingly higher than Spain and similar to Sweden.

Despite very poor staffing, Hungarian nurses provided the most positive perceptions about staffing for all countries, while Croatia was slightly less positive than Sweden with very good staffing. Croatian and Hungarian nurses were not consistently observed working in ‘crisis mode’ possibly due to fatigue, lack of motivation, or other issues. In this regard, Hungarian nurses have been reported to experience decreased professional motivation due to their authoritative supervisors, while high workloads and low wages contribute to the problem [64]. Croatian nurses usually work under higher emotional rather than physical demand [65], which can result in emotional exhaustion and burnout [66]. These characteristics can exacerbate the consequences of work overload as fatigue limits cognition and decreases work productivity [67,68]. Croatian and Hungarian nurses acknowledged the limited staffing but appeared to reconcile this as the reality for their practice environment.

Despite Croatian and Hungarian nurses perceiving patient care to be relatively safe and providing the most positive perspectives for the dimensions of feedback and communication about error and communication openness, they commented during interviews about authoritative leaders, poor communication, and punitive environments. For this reason, the survey perceptions seemed to be disconnected from the interview responses. While the Croatian nurses believed that their supervisors are punitive when mistakes are reported and felt afraid to speak freely, the Hungary nurses were unaware of mistakes in their units but believed there would be punitive consequences for the rare mistake.

In the Swedish context, nurses perceived communication as a patient safety concern due to the potential for missed transfers of information [69]. Swedish nurses were observed spending more time engaged in handovers and reviewing clinical records prior to beginning the shift. The nurses also have the lowest nurse-to-patient ratios and received compensation for this time. Although Spanish nurses are paid for their time, they were not always able to make the time to review clinical records due to the increased workload compared to Sweden. Nurses in Hungary and Croatia, however, are not compensated for the time required to complete handovers with a heavier workload.

Teamwork had the highest percentage of positive response in all countries except for Croatia. These results are similar to Spanish [57,70], Swedish [71,72], and Hungarian studies [46]. Croatia has a historical and cultural context for sharing common features with Hungary related to the geographical-political region as they transitioned into a post-socialist period. According to Sklebar et al. [73], the reluctance of health care professionals in Croatian hospitals to embrace a teamwork mentality is consistent with cultural norms. The tendency of Croatian units to function as a closed system is one of the main obstacles to establishing and maintaining a comprehensive patient safety system. Appearance rather than efficiency indicates deficiencies in the quality of the performance of the Croatian society, a factor that can influence to the dynamics within and between hospital units. Teamwork is an area that is frequently the focus of improvement, as 20 of the 33 intervention studies included training and communication initiatives in a safety culture systemic review report [74].

Fear to engage in open communication due to a punitive culture punishment results in under reporting errors and avoidable adverse events [75]. The nurses in Croatia, Hungary, and Spain believe that open communication was not present in their work environment. Previous research in Croatian reported that open communication is undesirable as questioning decisions or reflecting a contrary attitude is dangerous for professional stability [73]. For this reason, safety culture is limited by people concealing honest feelings, actual thoughts, and real attitudes [45]. The deficiencies in teamwork observed in the Croatian and Hungarian hospitals could be attributed to this cultural context. Authoritative supervision and hierarchical structures may result in the deep dissatisfaction with teamwork [73] in the context of the Croatian nurses in this study.

Supportive management practices focused on improving patient safety increases the frequency of events reported [76], and results in solutions to prevent reoccurrence. For this reason, executive walk rounds have been reported to be an effective strategy to increase positive interactions between staff and management [74]. Swedish studies indicate that the ability of an organization to improve by learning from mistakes is underdeveloped. When errors occur in the Swedish context, the problem-solving model does not function, resulting in less organizational learning and few system improvements [71,77]. Swedish nurses perceived the number of events reported to be low, indicating that there are unreported events. Previous studies have reported slightly higher results for this dimension [71,72]. Swedish nurses report errors when they occur; however, they recognize that notification is inconsistent, and depends on the type of incident and the work area [69]. 

Similar to this study, the previous work from Spain indicated that preventive measures are taken once an error occurs; however, these measures may not be monitored afterwards [57,61,78]. Also, there may be fear and distrust for engaging in incident notifications due to negative repercussions [78]. The non-punitive response to errors was one of the most negative findings in all countries except Sweden, which is one of the best rated in our study and in previous studies [72]. The non-punitive response characterizes a positive safety culture. This finding differentiates the safety cultural maturity [79,80] that characterizes Sweden in comparison to the other countries. The Croatian and Hungarian hospitals had a similar safety culture maturity which was less developed than Spain. 

### Limitations

In relation to the conceptual design of the study, data were collected in two hospitals in each participating country, which is not a representative number of the total number of public hospitals in each country. The generalizability of the findings, therefore, could be limited. The complexity of the study design did not result in similar quantitative sample sizes for each hospital or country. However, the sample included eight hospitals from four European Union, the first direct comparison of multiple countries using a mixed methods approach. Another potential limitation of this study was the focus on nurses, around 40% to 60% of the workforce in the hospitals. However, this is also a strength, as the nurses are the profession with the most front-line caregiving responsibilities in hospitals. The supervisor involvement in the data collection process could result in social desirability bias. Although this process was necessary, the surveys were deposited into a secured data collection box, accessible only to the primary investigator, in private locations on the nursing units. Finally, the sequential data collection process increases the potential for bias due to the time lag with each phase. The time lag is unavoidable when one investigator collects interview data and observes participant practices at multiple sites. However, the interviews and observations have less variability in recording and more uniformity in analysis.

## 5. Conclusions

This was the first multinational study reported in the literature that used a mixed method approach to measure patient safety culture and to compare hospitals in different countries. Despite all countries being members of the European Union, many safety culture dimensions were areas of weaknesses, with few dimensions defined as good, as most measured adequate to poor. Measured perceptions of safety culture at the dimension level were at times incongruent across countries with the observed patient safety practices or inconsistent with interview perspectives. Differences between countries may be related to national culture or variability in health system structures permitted by the prevailing flexibility of the European Union’s health policy. Safety culture should be regularly assessed in hospitals across the European Union to better understand the impact of national and regional variations on patient safety. Further research is necessary to understand the consistency of perceptions between countries and the stability of concepts defining safety culture across national borders in the European Union, as well as in global regions.

## Figures and Tables

**Table 1:** Positive perception (percentage) for each dimension by hospital and country.

Safety Culture Dimension	Country	Hospital 1	Hospital 2	Total	*p* between Hospitals within Country ^†^	*p* between Country ^†^
Frequency of event reporting	SW	36.5%	41.4%	38.1%	-	0.020 SP < HU
	SP	43.9%	42.8%	43.4%	0.885	
	HU	59.9%	54.3%	57.9%	0.451	
	CR	44.0%	50.0%	47.2%	0.609	
2. Overall perceptions of safety	SW	55.2%	37.3%	49.2%	-	0.000 SP < HU; SP < CR
	SP	31.0%	25.5%	28.7%	0.295	
	HU	59.8%	53.0%	57.6%	0.272	
	CR	50.9%	58.6%	54.9%	0.328	
3. Supervisor expectations and actions promoting safety	SW	62.1%	63.1%	62.4%	-	0.056
	SP	50.4%	45.7%	48.4%	0.468	
	HU	57.8%	55.9%	57.1%	0.689	
	CR	56.0%	60.5%	58.5%	0.654	
4. Organizational learning—continuous improvement	SW	52.0%	43.5%	49.1%	-	0.000 SP < HU; SP < CR
	SP	41.8%	43.5%	42.5%	0.800	
	HU	68.7%	59.2%	65.6%	0.100	
	CR	68.0%	55.9%	61.3%	0.166	
5. Teamwork within hospital units	SW	85.3%	77.7%	82.7%	-	0.002 CR < HU
	SP	70.2%	63.6%	67.4%	0.319	
	HU	74.1%	73.8%	74.0%	0.950	
	CR	69.4%	44.5%	55.9%	0.007	
6. Communication openness	SW	62.7%	60.1%	61.8%	-	0.708
	SP	55.6%	37.0%	47.7%	0.005	
	HU	47.6%	38.8%	44.6%	0.080	
	CR	45.8%	43.7%	44.7%	0.797	
7. Feedback and communication about error	SW	48.7%	55.9%	51.1%	-	0.000 SP < HU; SP < CR; CR < HU
	SP	27.0%	32.6%	29.4%	0.322	
	HU	73.4%	55.4%	65.8%	0.001	
	CR	50.7%	39.1%	44.2%	0.257	
8. Non-punitive response to error	SW	67.6%	53.5%	62.9%	-	0.048 CR < SP
	SP	39.7%	35.5%	37.9%	0.489	
	HU	42.2%	24.4%	36.3%	0.005	
	CR	19.2%	30.0%	25.0%	0.192	
9. Staffing	SW	39.1%	34.0%	37.4%	-	0.000 SP < HU; SP < CR
	SP	16.3%	11.4%	14.2%	0.112	
	HU	42.0%	37.8%	40.6%	0.465	
	CR	25.9%	38.3%	32.5%	0.033	
10. Hospital management support for patient safety	SW	36.3%	23.8%	32.1%	-	0.000 HU > CR; HU > SP
	SP	13.2%	24.6%	18.0%	0.055	
	HU	69.4%	47.2%	62.2%	0.002	
	CR	26.4%	18.9%	22.2%	0.349	
11. Teamwork across hospital units	SW	49.8%	44.0%	47.9%	-	0.013 CR < HU
	SP	45.6%	48.4%	46.8%	0.679	
	HU	52.1%	48.8%	51.0%	0.615	
	CR	45.0%	25.8%	34.5%	0.039	
12. Handoffs and transitions	SW	51.6%	57.3%	53.5%	-	0.225
	SP	54.4%	59.8%	56.7%	0.363	
	HU	54.1%	45.1%	51.2%	0.209	
	CR	63.0%	57.8%	60.2%	0.587	

^†^ Sweden is not included in the statistical analysis as item level by nurse data were not available.

**Table 2:** Positive perception (%) for each dimension by unit type within country.

Safety Culture Dimension	Country	Internal Medicine	General Surgery	Emergency Department	*p*-Value
Frequency of event reporting	SW	37.0%	39.0%	44.0%	-
	SP	53.9%	42.2%	38.8%	0.312
	HU	52.5%	63.2%	59.5%	0.396
	CR	58.3%	47.9%	33.3%	0.199
2. Overall perceptions of safety	SW	56.0%	35.0%	45.0%	-
	SP	26.9%	36.0%	24.5%	0.139
	HU	54.1%	57.8%	63.4%	0.479
	CR	61.5%	43.8%	56.3%	0.167
3. Supervisor/manager expectations and actions promoting safety	SW	53.0%	73.0%	72.0%	-
	SP	50.0%	48.5%	47.5%	0.953
	HU	59.0%	59.6%	49.2%	0.204
	CR	67.7%	45.0%	57.4%	0.170
4. Organizational learning—continuous improvement	SW	44.0%	43.0%	63.0%	-
	SP	51.3%	52.0%	31.3%	0.007 ^†^
	HU	65.3%	65.2%	66.7%	0.977
	CR	66.7%	57.1%	56.9%	0.545
5. Teamwork within hospital units	SW	80.0%	80.0%	86.0%	-
	SP	74.0%	83.1%	53.1%	<0.001 ^‡^
	HU	77.6%	70.4%	74.1%	0.463
	CR	57.0%	45.6%	64.7%	0.296
6. Communication openness	SW	54.0%	69.0%	69.0%	-
	SP	52.6%	52.9%	41.5%	0.235
	HU	43.1%	41.7%	52.4%	0.219
	CR	50.8%	41.7%	39.6%	0.480
7. Feedback and communication about error	SW	38.0%	57.0%	68.0%	-
	SP	35.9%	32.4%	23.8%	0.190
	HU	61.4%	67.9%	71.0%	0.380
	CR	47.6%	35.6%	47.9%	0.540
8. Non-punitive response to error	SW	66.0%	63.0%	57.0%	-
	SP	32.1%	44.1%	36.7%	0.308
	HU	38.8%	25.7%	50.6%	0.011 ^§^
	CR	26.7%	20.0%	27.1%	0.767
9. Staffing	SW	38.0%	18.0%	51.0%	-
	SP	12.5%	16.9%	13.3%	0.479
	HU	43.9%	36.2%	42.6%	0.414
	CR	35.4%	32.8%	27.9%	0.574
10. Hospital management support for patient safety	SW	34.0%	25.0%	29.0%	-
	SP	26.9%	15.7%	15.0%	0.191
	HU	60.5%	63.3%	63.1%	0.930
	CR	25.4%	27.5%	12.5%	0.276
11. Teamwork across hospital units	SW	56.0%	40.0%	37.0%	-
	SP	55.8%	44.1%	43.9%	0.304
	HU	57.7%	45.7%	48.2%	0.213
	CR	47.6%	27.9%	25.0%	0.083
12. Handoffs and transitions	SW	56.0%	50.0%	53.0%	-
	SP	64.4%	58.1%	51.5%	0.210
	HU	52.6%	49.5%	51.8%	0.919
	CR	66.7%	62.5%	50.0%	0.331

^†^ Tukey post-hoc contrast: differences between Emergency and Internal Medicine (*p* = 0.035) and between Emergency and General Surgery (*p* = 0.015). ^‡^ Tukey post-hoc contrast: differences between Emergency and Internal Medicine (*p* = 0.021) and between Emergency and General Surgery (*p* < 0.001). ^§^ Tukey post-hoc contrast: differences between Emergency and General Surgery (*p* < 0.005).

## Data Availability

The quantitative data is available from the corresponding author upon a reasonable request. Defined segments of the qualitative data may also be available upon a reasonable request if the anonymity and confidentiality of the participants and the hospitals can be assured through the data release.
